# Craig Venter, a microbiologist

**DOI:** 10.1128/mbio.01732-26

**Published:** 2026-09-18

**Authors:** Rino Rappuoli

**Affiliations:** 1 Fondazione Biotecnopolo di Siena 840017 , Siena, Italy; Georgia Institute of Technology, Atlanta, Georgia, USA

**Keywords:** Craig Venter, vaccinology, synthetic biology, microbiology, vaccine development

## Abstract

The scientific career of Craig Venter, who pioneered genomics, big data, and synthetic biology, has been a turning point in biology. His science will continue to influence the world forever. He will be remembered for the first genome of a living organism, for the human genome, and for the first fully synthetic bacterial chromosome. I was fortunate to walk part of an amazing journey with Craig and experience first-hand what visionary science is and what it can lead to for the benefit of humankind. In this article, I make the case that Craig should be remembered also for his revolutionary contribution to microbiology and vaccinology. Indeed, he was the first to sequence a bacterial genome and the first to get a glimpse of the almost infinite diversity of the microbial world, and he pioneered the transition from conventional to modern vaccinology by introducing genomics and synthetic biology.

## COMMENTARY

The death of Craig Venter from a sudden cancer has generated several commentaries and obituaries in important scientific journals to honor the revolutionary innovations he brought to life sciences (1–3). Craig’s achievements included the first genome sequence of a living organism, the sequence of the human genome, and the first synthetic cell, as well as the controversial interactions with the official science that accused him of using the information for commercial purposes. In this commentary, I would like to describe another important but often neglected impact that Craig has had on the well-being of humanity by contributing to the beginning of a new era in microbiology and vaccinology. Specifically, I will focus on three aspects: his role in the understanding of the nearly infinite diversity of microbes that populate the oceans of our planet; his contribution to vaccinology and the beginning of the era of reverse vaccinology; and, finally, the advent of synthetic biology and the development of vaccines in 1 week.

## FROM BACTERIAL GENOMES TO IMMENSE DIVERSITY OF THE PLANETARY MICROBIOME

The first contribution of Craig Venter to microbiology was the sequence of the genome of the *Haemophilus influenzae*, a milestone in the history of science, since this is the first sequence of the genome of a living organism. However, this was just the beginning of his contribution to microbiology. Craig was passionate about two things—science and sailboats—and he decided to combine them using his sailboat to do science. In 2003, he decided to sequence the genome of the water of the Sargasso Sea, making the incredible discovery that 200 L of water contained 1,800 genomic species, 148 previously unknown bacterial phylotypes, and 1.2 million previously unknown genes (4). Excited by this incredible discovery, between 2003 and 2018, Craig navigated the oceans, starting from the Galápagos Islands, following the footsteps of Charles Darwin. His quest was to discover the nature and evolution of microbes, those invisible living organisms that were too small to capture Darwin’s attention. The world unveiled by Craig went beyond imagination: a trillion microbial species, each with its own pangenome, containing millions of related but different microorganisms. This was quite different from the relatively small number of animals that Darwin had to deal with. Even the availability of supercomputers could not help Craig draw the lines of microbial evolution because the diversity was in all directions and in all environments without obvious rules, other than using horizontal transfer, phages, transposons, and plasmids to capture the genes that would fit each of the infinite surroundings. The story is well described in the book *The Voyage of Sorcerer II* (5), which also reveals some interesting adventures dealing with the local authorities of many states that were hesitant to grant permission to sample the waters of their environment because they were afraid that Craig would extract their secrets. The overall conclusion of the Sorcerer II journey is that the microbial universe is much larger than we have ever imagined; it has incredible diversity and performs functions needed for life and our planet. Indeed, Craig started the discovery of the fascinating and mostly infinite universe of the microbes that populate our oceans.

## REVERSE VACCINOLOGY

In the mid-1990s, my research group at Chiron Corporation had failed to find a solution to make a vaccine against serogroup B meningococcus, a bacterium that caused 50% of the global cases of a devastating disease that can kill healthy babies and adolescents in less than 24 hours. Our group and other vaccine developers worldwide had been using all known technologies available at that time to find a solution. However, we failed because of the ability of the bacterium to generate variants exposing almost an infinite diversity of antigens and its ability to evade our immune system by expressing antigens mimicking those present on our cells. All conventional technologies and contemporary science were unable to design a vaccine against this bacterium. It was clear that the solution required a revolutionary approach.

In the summer of 1995, when Craig published the genome of the bacterium *H. influenzae*, the world commented on the revolutionary technology that, for the first time, had revealed the blueprint of a living organism. Our group recognized that by sequencing the genome of meningococcus B, we would have the opportunity to discover all the antigens encoded in the genome and find the protective antigens that were missed by conventional technologies.

With this in mind, I visited Craig at The Institute for Genomic Research (TIGR) in Rockville. I had never met him before, but I got a glimpse of his personality while walking through the corridor at TIGR that led to his office. The walls were packed with pictures of Craig and newspaper clippings about him. At the end of the corridor was his office, where he sat behind a very nice desk with a dog crouched on the floor next to it. Craig was very friendly, but when I told him that I wanted to sequence the genome of a bacterium, he replied that it was not in his plans to sequence another bacterial genome. He had already sequenced one, and the two million-base-pair genome had been important for setting up the software and the hardware needed to sequence genomes. His next challenge was, however, to do the 180 million-base-pair genome of the *Drosophila* fruit fly to shape the technology and approach the 3.3 billion base pairs of the human genome. He was determined with this plan, but I did not give up. I told him that I clearly understood his ambition, but I also explained that there was a different reason to sequence the genome of meningococcus B: the possibility to develop a vaccine that could save the lives of babies and adolescents.

Fifteen minutes later, Craig had understood the importance of the project and committed to sequencing the genome, charging only the live costs of the sequence. The deal was defined, and we started a collaboration involving TIGR to sequence the genome; Richard Moxon, a pediatrician and geneticist from the University of Oxford with profound knowledge of genomes; and our group in Siena, led by Mariagrazia Pizza, to transform that genome knowledge into vaccines. In 2000, this collaboration resulted in the publication in *Science* of two milestone papers: one on the genome of meningococcus B and the other on the identification of previously unknown antigens that could be the basis of a vaccine that otherwise was impossible to develop (6, 7). We named this new approach to make vaccines “reverse vaccinology” (8) because instead of starting from the cultivated pathogens, we started from the computer, accessing all the information contained in the genome. This was the first time that “big data” were used to develop a pharmaceutical product. Following extensive pre-clinical and clinical studies, the meningococcus B vaccine was licensed in Europe in 2013 and in the United States in 2015. The vaccine is now administered in more than 50 countries. Today, no bacterial or viral vaccines are developed without knowing the genomic sequence of the pathogen, and reverse vaccinology has become a standard approach for vaccine discovery, including sequencing the genome of tumors to design personalized cancer vaccines (Fig. 1).

**Fig 1:**
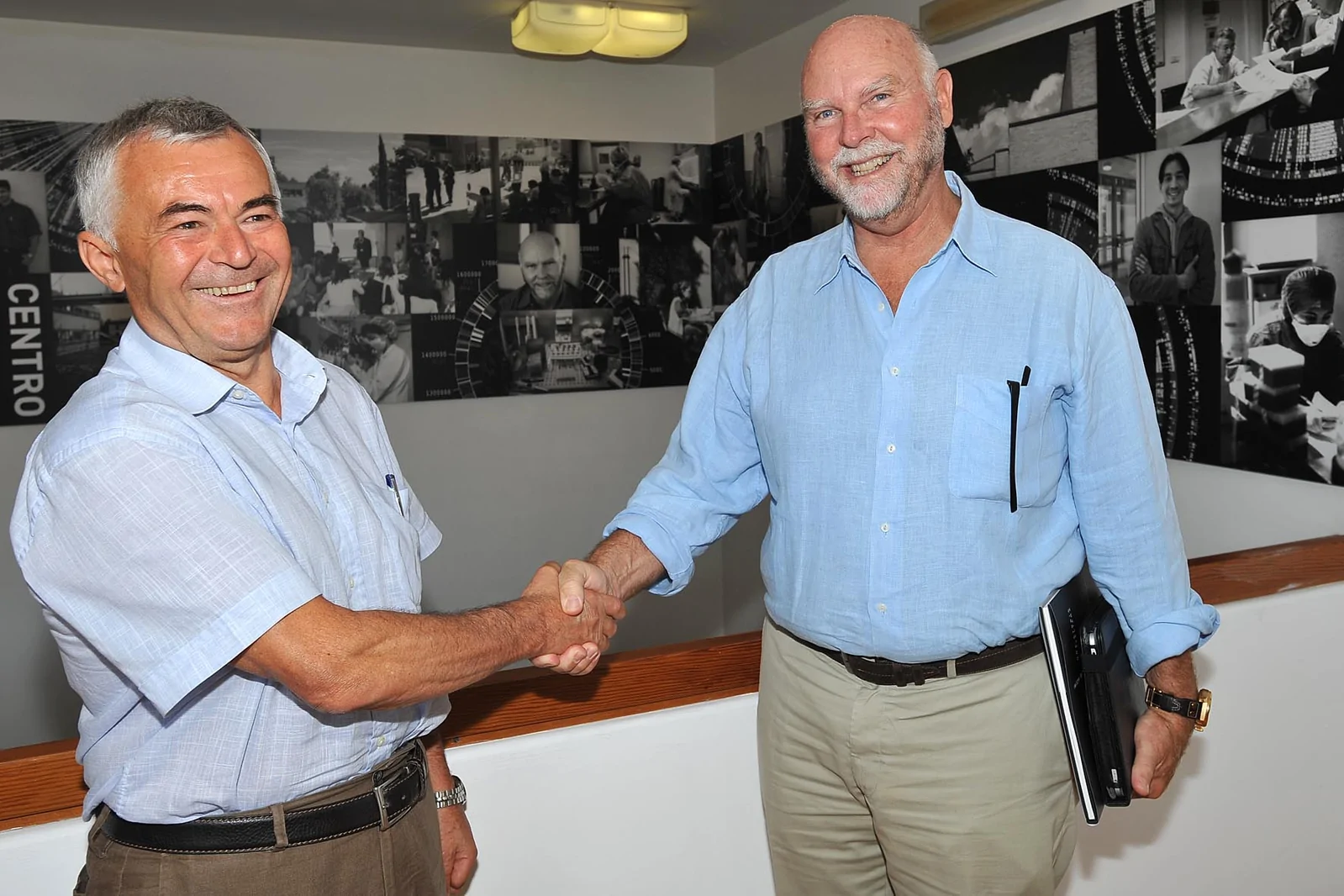
Craig Venter visiting Rino Rappuoli in Siena, Italy (2010).

## SYNTHETIC BIOLOGY AND VACCINES IN 1 WEEK

Following the nice collaboration on the meningococcus B vaccine, my team used to visit TIGR to exchange our progress. During one of these visits, Craig informed me that he was focusing on synthetic biology to generate new life and suggested that I should be thinking about how to use that new technology he was pioneering to make vaccines. Initially, I had no idea how synthetic biology could be useful to make vaccines, but when we met the following year, we had matured the vision that we could use it. For instance, through this technology, we could quickly make synthetic genes coding for the hemagglutinin (HA) and the neuraminidase (NA) of the influenza virus so that we could rapidly make vaccines against pandemics of avian influenza or seasonal influenza. We decided to submit a grant application to BARDA, promising to make a seed for an influenza vaccine in less than a month after receiving from BARDA the HA and NA sequences. Thanks to the formidable ability of TIGR to generate correct synthetic genes, we successfully generated the viral seed in less than 1 month. The opportunity for the real demonstration of the transformative power of synthetic biology came on Sunday, 31 March 2013, which coincided with Easter Day. The Chinese Center for Disease Control had posted on its website the sequence of the HA and NA genes of an avian H7N9 virus that had killed three people in China and had all the properties to cause a pandemic. Since making a conventional vaccine required several months to get the virus from China and adapt it to grow in eggs, we decided to take a different approach and start from a synthetic gene. On Monday, Craig synthesized the genes coding for the HA and NA genes. These were sent by express mail so that on Tuesday morning, they could reach the laboratories of Novartis in Cambridge, MA. As soon as the genes arrived, our team started using the synthetic genes to generate a virus that could be applied as a seed to make a conventional vaccine, and in parallel, the synthetic genes were used to make an mRNA vaccine. Remarkably, both vaccines were ready in 1 week, months before the real virus was available for manufacturing of conventional vaccines (9, 10). This was the first time a vaccine was produced in less than 1 week using the information from the internet, without the need for the real virus. Fortunately, the H7N9 virus did not cause a pandemic, but the methodology we used in 2013 to make a vaccine against avian influenza was exactly the one replicated on 10 January 2020, when the sequence of the SARS-CoV-2 virus was posted on the internet by the Chinese Center for Disease Control. More than 300 labs worldwide downloaded the sequence, synthesized the gene coding for the Spike protein, and used it to make synthetic mRNA vaccines, to engineer viral vector vaccines, and to engineer mammalian, insect, or plant cells to make a recombinant spike protein, without the need to even see the virus.

In trying to find an appropriate conclusion to my tribute to Craig Venter’s scientific contributions and to show how these have been a game changer for research and development, I cannot but wonder how he would enjoy seeing how his pioneering work in genomics and synthetic biology, propelled by AI technologies, is going to shape the future of microbiology, science, and humanity. I can only thank him for what he has done to make all this possible.
